# Case report of a pseudo‐isodicentric chromosome 9 resulting in mosaic trisomy 9

**DOI:** 10.1002/ccr3.4031

**Published:** 2021-03-09

**Authors:** Sarah M. Beaudry, Oleg Shchelochkov, Pamela Trapane, Benjamin Darbro, Jaime M. W. Nagy

**Affiliations:** ^1^ Division of Medical Genetics and Genomics Department of Pediatrics University of Iowa Hospitals and Clinics Iowa City IA USA; ^2^ Medical Genomics and Metabolic Genetics Branch National Human Genome Research Institute National Institutes of Health Bethesda MD USA; ^3^ Division of Pediatric Genetics Department of Pediatrics UF College of Medicine–Jacksonville Jacksonville FL USA

**Keywords:** long‐term survival, mosaic trisomy 9, pseudo‐isodicentric chromosome 9

## Abstract

Due to the variable presentation of mosaic chromosomal abnormalities, cases such as this are needed to define the phenotypic spectrum. It also highlights the importance of chromosome analysis to identify structural abnormalities that result in aneuploidy.

## Graphical abstract

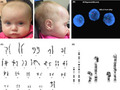

## 1 INTRODUCTION

Mosaic trisomy 9 cases are rare, presenting with varying phenotypes and degrees of severity. We describe a patient with mosaic trisomy 9 from a pseudo‐isodicentric chromosome 9 with features similar to previously reported trisomy 9 patients. To our knowledge, this is the first reported case of a pseudo‐isodicentric chromosome 9.

Complete trisomy 9 is rare in live births (0.01%)^1^ and is the cause of 2.4% of studied spontaneous abortions.^2^ Mosaic trisomy 9 is the 5th most common trisomy detected in live births.^3^ Compared to full trisomy 9, mosaic trisomy 9 may be associated with longer survival, and some patients live into adulthood.^4^ This trisomy affects males and females equally and is found among all ethnicities. The first clinical case was described in 1973,^5^ and over the years, more than 50 cases have been described. Affected individuals can vary in the degree of their manifestations, the severity of organ involvement, and neurocognitive outcomes. Long‐term outcomes have been described infrequently. In this case report, we present a four‐year follow‐up of a patient affected by a mosaic pseudo‐isodicentric trisomy 9.

## 2 CASE REPORT

The patient was to born to nonconsanguineous parents in their early thirties at 38 6/7 weeks gestation via caesarian section, due to vaginal bleeding and breech positioning. This was the mother's third pregnancy, and the couple has two older healthy children. A three‐generation pedigree was negative for any known genetic diseases, chromosomal abnormalities, multiple miscarriages, and infertility. The pregnancy was uncomplicated until the seventh month of gestation, in which the mother was diagnosed with Guillain‐Barre syndrome and was treated with plasmapheresis. At the same time, the mother also had a positive cytomegalovirus (CMV) PCR.

**FIGURE 1:**
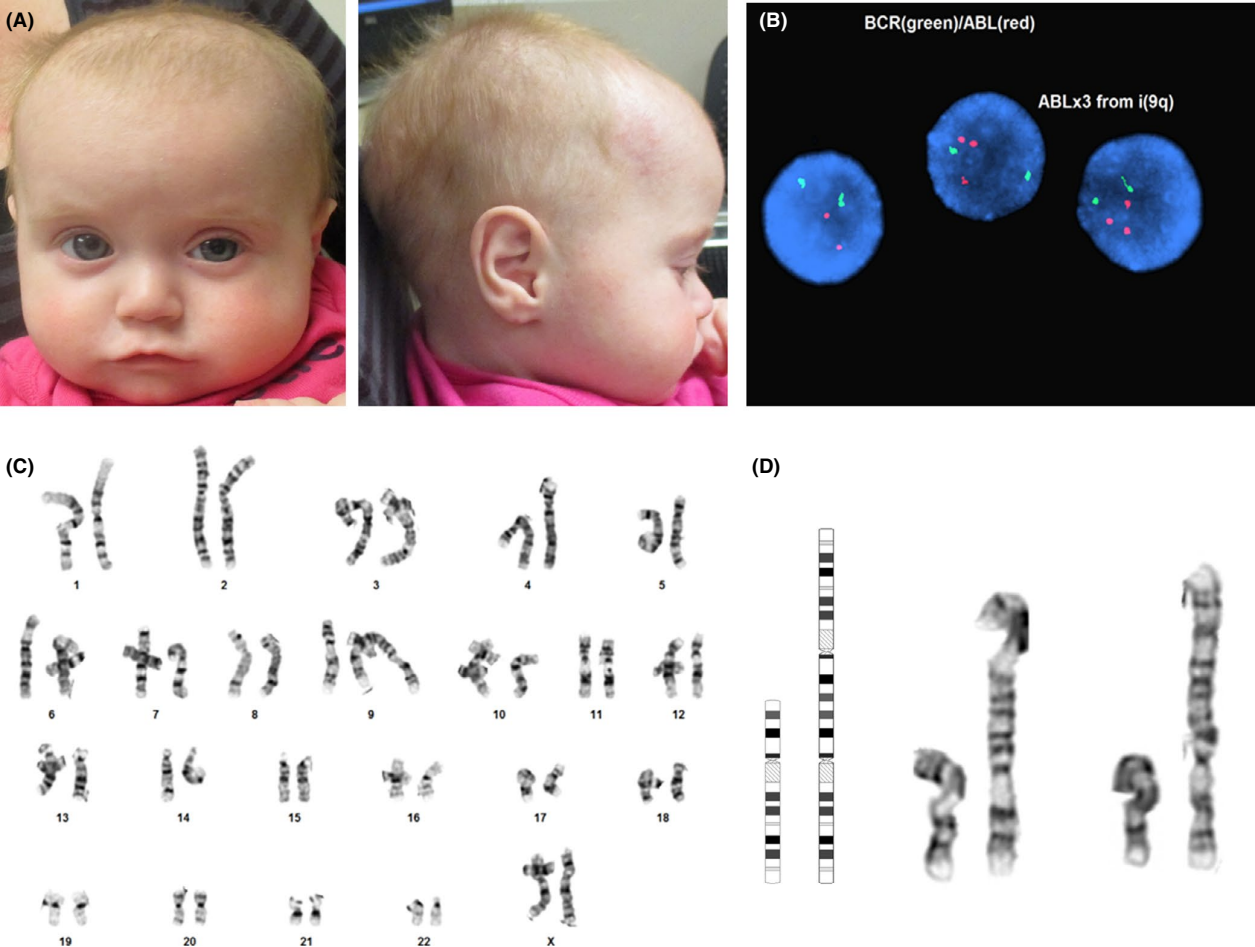
(a) Frontal and profile views of patient at 7 months of age. (b) Interphase FISH showing the gain of ABL (red) due to the pseudo‐isodicentric 9. (c) G‐banded karyotype of patient's pseudo‐isodicentric 9. (d) Ideogram and partials of patient's pseudo‐isodicentric chromosome 9

At birth, the patient weighed 3.57 kg (76%tile), was 50 cm long (68%tile), and had a head circumference of 35.3 cm (80%tile). Apgar scores were 7 at one and 8 at five minutes. Respiratory distress required supplemental oxygen via nasal canula until the ninth day of life. She had hypotonia, feeding difficulties, and laryngomalacia type I. An echocardiogram demonstrated a patent ductus arteriosus (PDA). Thrombocytopenia on day of life 1 was 83 000/mm^3^ and improved to 112 000/mm^3^ on the 58th day of life. Transient hypoglycemia resolved by the ninth day of life. Dysmorphic features included narrow palpebral fissures, tall forehead, thin upper lip, smooth philtrum, depressed nasal bridge, small ears, and small hands (Figure 1A). A dilated eye examination did not reveal any structural abnormalities. There was no evidence of dental, kidney or urinary, musculoskeletal, or skin abnormalities, and her newborn screen was normal. A quantitative PCR test on blood and urine samples was negative for congenital CMV.

Chromosome analysis revealed mosaicism for a pseudo‐isodicentic chromosome 9 with a breakpoint at 9p24 in 5 out of 20 metaphase cells (25%) [mos 46,XX,psu idic(9)(p24.3)[5]/46,XX[15]] (Figure 1C, D). Almost all of chromosome 9 was duplicated in this rearrangement (as 9p24.3 is the terminal short‐arm band), resulting in a mosaic trisomy 9, where one of the centromeres appeared to be inactive leading to a stable dicentric chromosome. Fluorescent in situ hybridization (FISH) was performed using the DNA probe for BCR/ABL (22q11.2/9q34). These results showed three ABL (9q34) signals in 17% of the nuclei, (nuc ish(ABLx3,BCRx2)[51/300]), thus confirming the presence of three copies of the long arm of chromosome 9 (Figure 1B). Chromosomal microarray and subtelomere FISH were not preformed. Therefore, we cannot rule out a submicroscopic terminal deletion of 9p on the isodicentric chromosome. By chromosome banding, the isodicentric chromosome appears to contain all p‐arm material up to the terminal 9p24.3 band. Terminal 9p24.3 deletions have been associated with gonadal dysgenesis and 46,XY disorders of sexual development.^6^ However, a mosaic deletion of this region, if present, would be unlikely to contribute to our patient's phenotype. Parental chromosome and FISH studies were not obtained to determine whether the origin of the pseudo‐isodicentric chromosome 9 was inherited or de novo.

Early infancy was complicated by feeding difficulties due to her hypotonia and laryngomalacia. Therefore, on the 56th day of life, a G‐tube was placed and then replaced with MIC‐Key gastrostomy device at 5 months of age until the patient was 14 months old. Her hearing test at 20 months of age was normal. She was also diagnosed with acquired hypothyroidism, which continues to be managed with 50mcg of levothyroxine.

Developmental delay was noted, in the patient, at 14 months when her gross motor skills were that of a 6‐ to 7‐month‐old. Her speech, receptive language, and expressive language skills were that of a 7‐ to8–month‐old, and her fine motor skills were at a 12‐ to 14‐month‐old level. At 20 months, the patient was able to scoot from one place to another and sit without support. Her gross motor skills were that of a 9‐ to 10‐month‐old, fine motor skills at 14 months, speech skills at an 8‐month‐old level, and language skills at about 12 months. She also understood the meaning of the word “no” and played well with her siblings. At 23 months, she was able to pull herself up to a standing position. At 2 years and 3 months, she was cruising, walking with assistance, and had about 40 words in her vocabulary, along with making friends at daycare. By 2 years and 10 months of age, she was able to walk independently and was verbalizing even more. She continues to interact well with her siblings and the family dog. At 4 years 6 months of age, she has short stature, continues treatment for hypothyroidism, and has some global developmental delay but is attending preschool (Supplemental Figures S1, A‐D and Supplemental Table S1).

## 3 DISCUSSION

Clinical features of mosaic trisomy 9 can vary in their presentation and severity, making them difficult to predict.^7^, ^8^ In almost all patients with mosaic trisomy 9, some degree of developmental delay has been described. These vary in severity, with fine and gross motor delays being the most prevalent, followed by speech and language delays.^8^, ^9^ In addition, one of the cases reported by Zen et al (2010) presented with swallowing problems, gastroesophageal reflux, and significant hypotonia.^10^ Several other children have been described to exhibit difficulty feeding as newborns and some had a tube placed to aid in their nourishment^8^, ^9^, ^11^, ^12^ (Table 1). Our patient's clinical findings of hypotonia, laryngomalacia, feeding difficulties, and short stature have been reported in several other trisomy 9 cases.^8^, ^9^

**TABLE 1:** General Characteristics of Trisomy 9

	Our Patient	Pejcic et. al.	Zen et. al. P1	Zen et. al. P2	Sanchez et. al.	Santo et. al. P1	Santo et. al. P2	Bruns and Campbell (out of 25 patients)	Prevalence of findings
Short Stature	X	X	X	X					4/32 (12.5%)
Hearing Loss				X					1/32 (3.12%)
Language delay	X	X	X	X		X	X	Many	>50%
Fine/gross motor delay	X	X		X		X	X	Many	>50%
Abnormal Facial features	X	X	X	X	X		X	16	22/32 (68.75%)
Cardiac Anomalies^a^	X	X		X		X		11	15/32 (46.88%)
Respiratory issues	X	X							2/32 (6.25%)
Feeding Issues	X	X		X	X	X	X	19	25/32 (78.12%)
Kidney abnormalities								5	5/32 (15.63%)
Muscular/Skeletal anomalies	X	X			X			2	5/32 (15.63%)

^a^ Cardiac anomalies include arterial septal defect (ASD), ventricular septal defect (VSD), patent ductus arteriosus (PDA), and aberrant left subclavian artery (ASA).

Although clinical findings in this case are consistent with mosaic trisomy 9, the pregnancy was complicated by CMV infection in the 3rd trimester, increasing the risk of congenital CMV infection. Some of the symptoms overlap with other reported cases of mosaic trisomy 9 patients; however, our patient repeatedly tested negative for CMV, supporting the pseudo‐isodicentric chromosome 9 as the cause of her phenotype.

Isodicentric and pseudo‐isodicentric chromosomes are rare constitutional abnormalities in humans. Usually, the formation of these derivatives causes a partial monosomy and partial trisomy of the chromosome(s) involved, which is rarely compatible with life. There are two general processes by which these isodicentric chromosomes are thought to form. The first process takes place during either the S or the G_2_ phase of the cell cycle in either mitosis or meiosis, where a break occurs in both chromatids followed by rejoining of the broken ends (referred to as an isolocal U‐shaped sister chromatid exchange) resulting in an isodicentric chromosome.^13^ In the second scenario, during G_1_ phase of the cell cycle, a small terminal deletion is followed by a U‐shaped rejoining of the broken chromatids, or “sticky” chromatids, after the replication of the deleted portion, that forms the isochromosome.^14^ Due to the mosaic nature of the abnormality seen in this patient, the isochromosome likely arose from a mitotic error which has been proposed for other mosaic autosomal isochromosomes.^15^ However, the exact mechanism resulting in isochromosome formation in this patient is unknown.

## 4 CONCLUSION

Mosaic trisomy 9 cases are rare and present with varying phenotypic features and degrees of severity. To our knowledge, this is the first report of a mosaic pseudo‐isodicentric chromosome 9. The patient's symptoms were consistent with those of other mosaic trisomy 9 cases, including a PDA, narrow palpebral fissure, small ears, hypotonia, and hip dysplasia all present at birth, and currently short stature and global developmental delay. Due to the highly variable presentation of mosaic trisomy 9, additional cases are needed with long‐term follow‐up to fully understand the phenotypic spectrum.

## CONFLICT OF INTEREST

None declared.

## AUTHOR CONTRIBUTIONS

SB: analyzed data and wrote manuscript. OS and PT: clinically evaluated the patient and reviewed the manuscript. JN and BD: analyzed and interpreted data and reviewed the manuscript.

## EDITORIAL POLICIES AND ETHICAL CONSIDERATIONS

Parental consent was obtained for publication of patient photographs.

## Supporting information

Fig S1A

Fig S1B

Fig S1C

Fig S1D

Table S1

Supplementary Material

## Data Availability

Data sharing not applicable to this article as no datasets were generated or analyzed during the current study.
