# Supplementary figures and images for: Case report of a pseudo‐isodicentric chromosome 9 resulting in mosaic trisomy 9
Source: Clin Case Rep. 2021 Mar 9;9(4):2340–4. doi: 10.1002/ccr3.4031 (PMC8077311; doi:10.1002/ccr3.4031)

**Growth Chart: United States**  
**Weight-for-age Percentiles (Girls, birth to 36 months)**

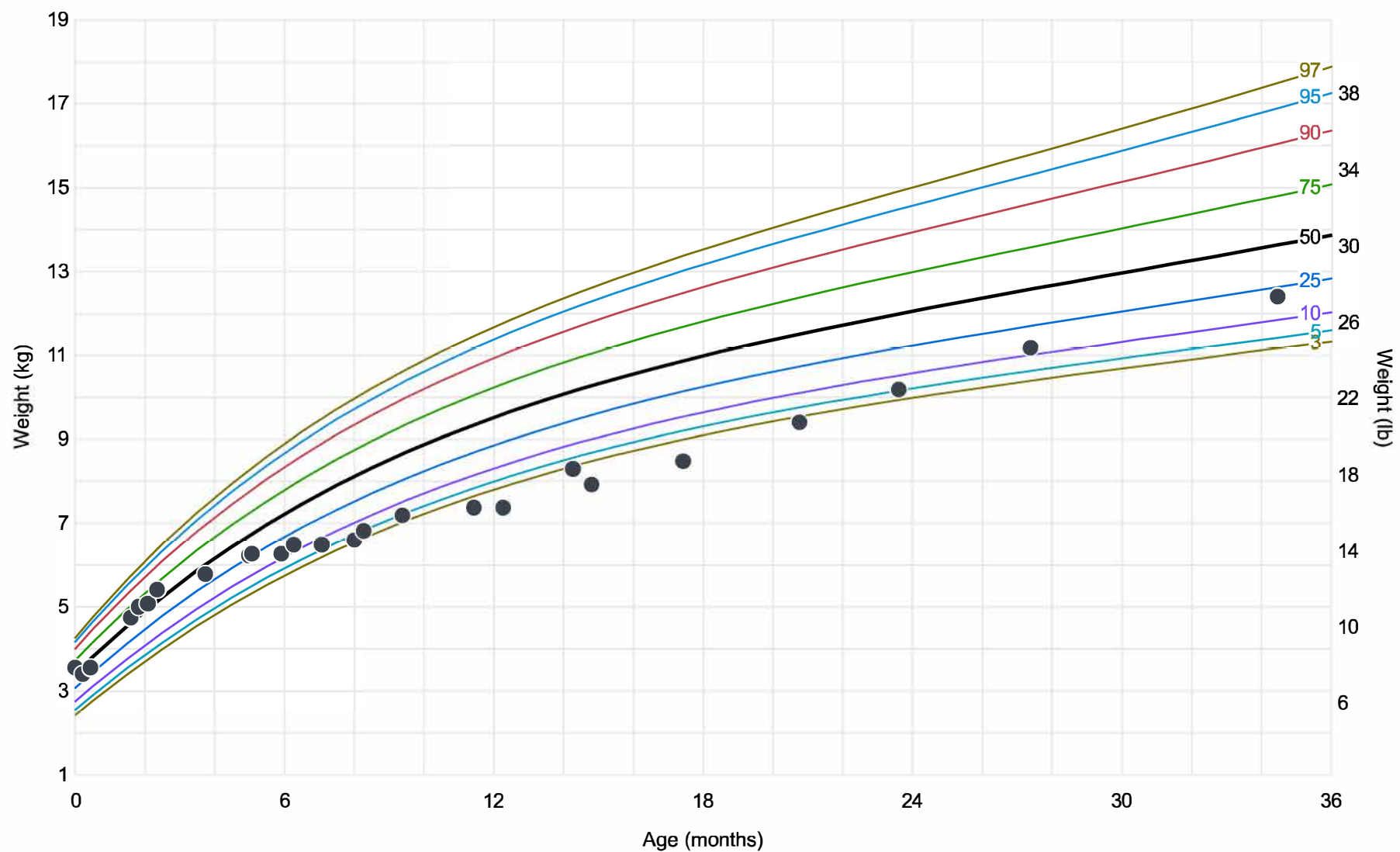

Supplement: Supplementary file 1 — Fig S1A [file CCR3-9-2340-s003.pdf]

**Growth Chart: United States**  
**Length-for-age Percentiles (Girls, birth to 36 months)**

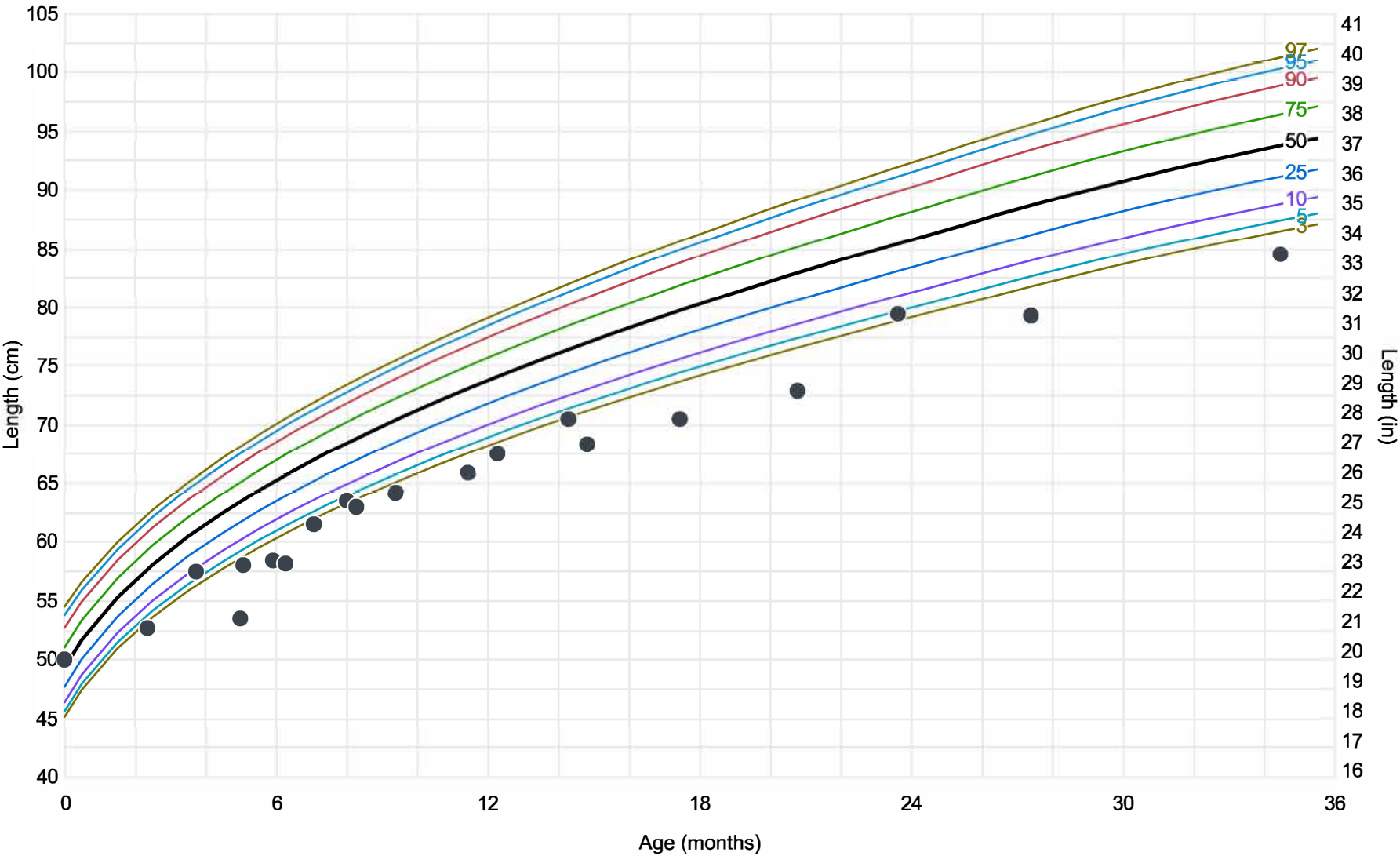

Supplement: Supplementary file 2 — Fig S1B [file CCR3-9-2340-s005.pdf]

**Growth Chart: United States**  
**Weight-for-age Percentiles (Girls, 2 to 20 years)**

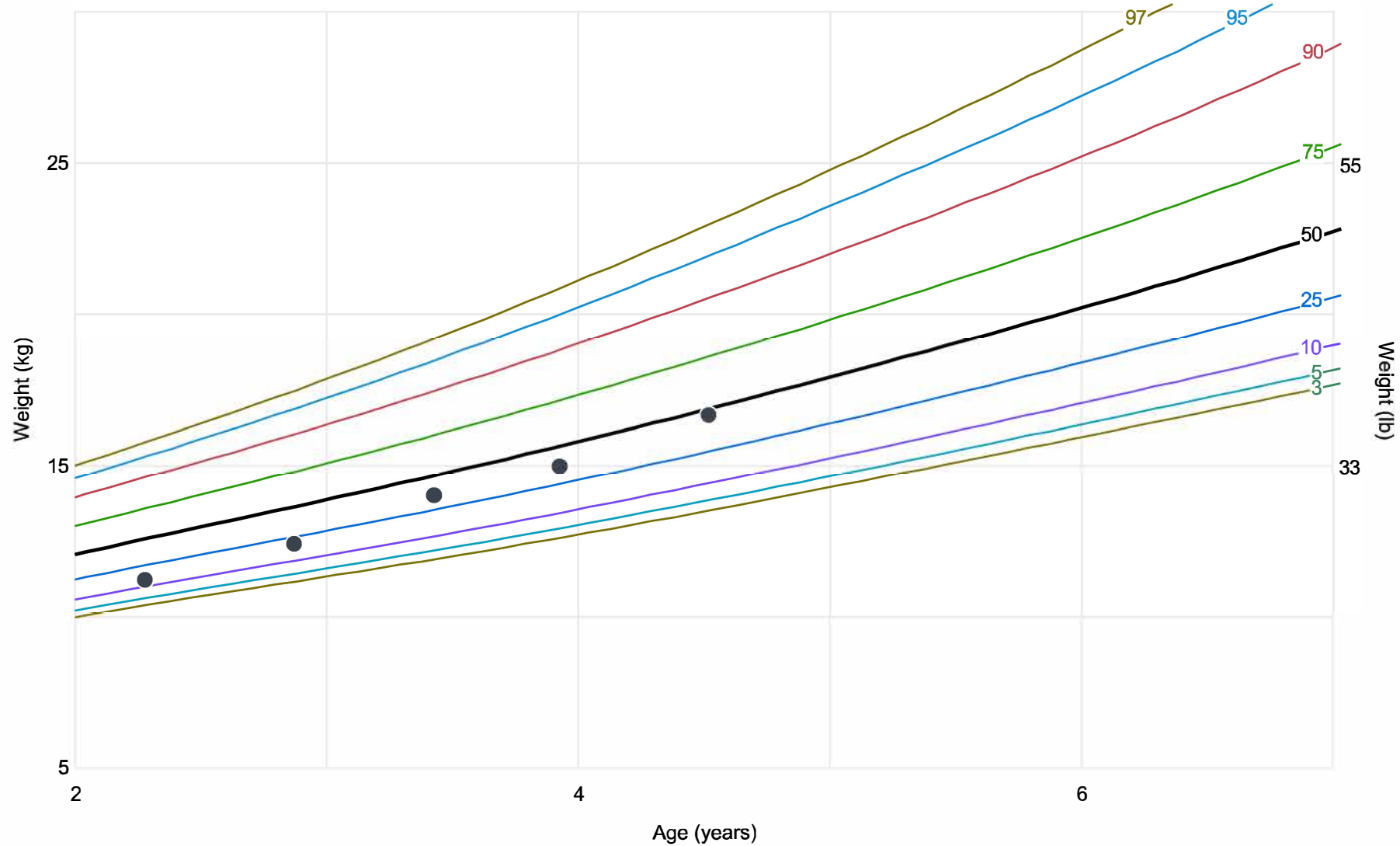

Supplement: Supplementary file 3 — Fig S1C [file CCR3-9-2340-s001.pdf]

**Growth Chart: United States**  
**Stature-for-age Percentiles (Girls, 2 to 20 years)**

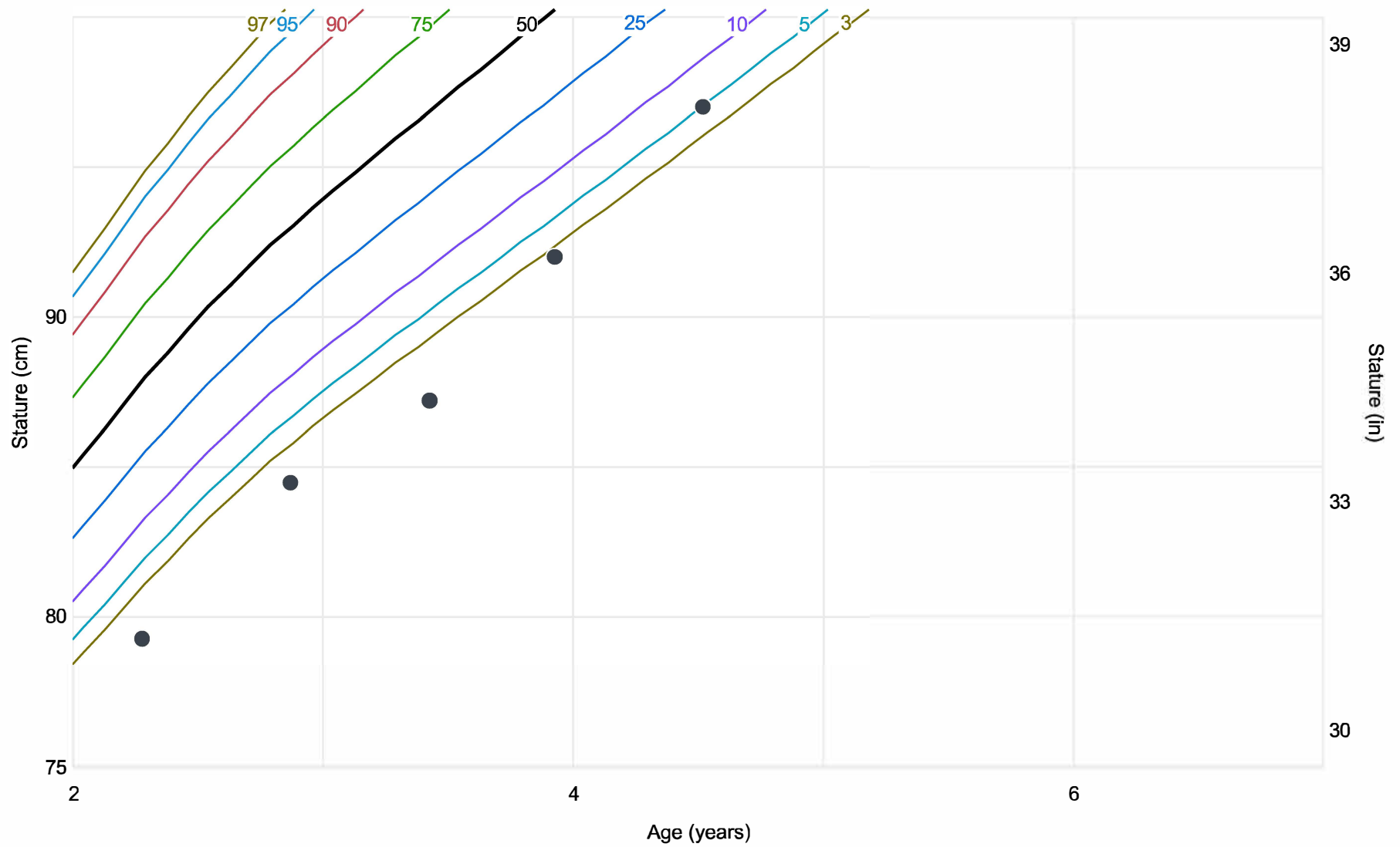

Supplement: Supplementary file 4 — Fig S1D [file CCR3-9-2340-s002.pdf]
