# Supplementary material for: Case report of a pseudo‐isodicentric chromosome 9 resulting in mosaic trisomy 9
Source: Clin Case Rep. 2021 Mar 9;9(4):2340–4. doi: 10.1002/ccr3.4031 (PMC8077311; doi:10.1002/ccr3.4031)
Supplement: Supplementary file 6 — Supplementary Material [file CCR3-9-2340-s004.docx]

**Supplemental Figures and Tables:**

**Supplemental Figure 1:**

A: Weight growth chart of patient from birth to 3 years (36mo.) of age

B: Height growth chart of patient from birth to 3 years (36mo.) of age

C: Weight growth chart of patient since 2 years of age

D: Height growth chart of patient since 2 years of age.

**Supplemental Table 1:** Height and Weight of Patient over time;

Footnotes: ‡Growth and Weight Z scores based on WHO (Girls, 0-2 years) data;

§Growth and Weight Z scores percentiles based on CDC 2-20 years data
